# Perceived community environment and depressive symptoms among older adults in China: a cross-sectional analysis of physical activity as a moderator

**DOI:** 10.3389/fpsyg.2026.1893833

**Published:** 2026-08-24

**Authors:** Zhijia Du, Junyue Yue, Zirui Lin, Xiaoyu Han

**Affiliations:** 1 Department of Social Welfare, Jeonbuk National University, Jeonju, Republic of Korea; 2 College of Business Administration, Guangdong Baiyun University, Guangzhou, China; 3 College of Physical Education, Dalian University, Dalian, China

**Keywords:** age-friendliness, depressive symptoms, older adults, perceived community environment, physical activity

## Abstract

**Introduction:**

Perceived community environment is an important contextual factor related to depressive symptoms in later life. However, few studies have examined whether physical activity (PA), a modifiable behavioral factor, moderates this association, particularly across specific aspects of perceived community environment and different types of PA.

**Methods:**

This study used data from the 2023 China Longitudinal Aging Social Survey, including 9,410 older adults. Linear regression models were used to examine the association between perceived community environment and depressive symptoms and to test the moderating effect of PA. Additional analyses examined specific aspects of the perceived community environment and different PA, including inactive, low PA, moderate PA, and high PA.

**Results:**

Poorer perceived community environment was associated with higher depressive symptom scores, and PA significantly moderated this association, although the incremental explanatory value was small (Δ adjusted *R*^2^ = 0.001). Compared with the inactive group, the perceived community environment slope was weaker in the low PA (*B* = −0.04, 95% CI: −0.07 to −0.001), moderate PA (*B* = −0.08, 95% CI: −0.12 to −0.03), and high PA (*B* = −0.26, 95% CI: −0.50 to −0.02). In community-clustered and weighted sensitivity analyses, the omnibus interaction was no longer statistically significant, although the interaction between perceived community environment and moderate PA remained statistically significant. After Holm correction, significant item-level interactions remained for perceived safety, cleanliness, street lighting, and competence of community staff.

**Conclusion:**

This cross-sectional study found that poorer perceived community environment was associated with higher depressive symptom scores among older adults in China, and that this association varied across different PA and specific aspects of community environment. These findings highlight the importance of older adults’ own evaluations of their communities and suggest that community-based mental health promotion may benefit from combining improvements in community conditions with accessible and age-appropriate PA opportunities, rather than treating PA as a substitute for environmental improvements. Given the modest improvement in model fit and the cross-sectional design, these findings should be confirmed in longitudinal and intervention studies.

## 1 Introduction

Population aging has made mental health in later life an increasingly important public health and social policy concern. China is experiencing rapid population aging and has one of the largest older populations in the world (Nie et al., 2021). As population aging accelerates, depressive symptoms among older adults have become a particularly salient issue because of their high prevalence and wide-ranging consequences. A recent meta-analysis estimated that approximately 20.0% of older adults in mainland China experience depressive symptoms (Tang et al., 2021). Depressive symptoms in later life are associated with poorer functional performance, reduced independence, lower social participation, and diminished quality of life (Liang, 2024; Sivertsen et al., 2015). They may also increase caregiving demands on families and impose sustained pressure on health and social service systems (GBD 2019 Mental Disorders Collaborators, 2022; König et al., 2019; Reynolds et al., 2022). Identifying modifiable and contextually relevant factors associated with depressive symptoms is therefore important for promoting psychological well-being and healthy aging among older adults (Chen et al., 2022).

The community environment is increasingly recognized as an important contextual factor related to mental health in later life (Nan et al., 2025; Wu et al., 2025). Compared with younger adults, older adults often have more spatially constrained daily lives and may rely more heavily on their immediate residential communities for daily activities, social interaction, access to services, and health-related behaviors (Nan et al., 2025). This is especially relevant in China, where home- and community-based eldercare has become an important component of aging policy and service provision (Krings et al., 2022; Xu et al., 2023; Ying et al., 2024). In this context, the community is not merely a physical place of residence; it is also a key setting through which older adults maintain social connections, obtain support, and interact with local service systems. Poor community environments may be associated with increasing daily stress, restricting social participation, reducing perceived safety and convenience, and weakening older adults’ sense of belonging and control (Lin et al., 2024; Lu et al., 2022; Mao et al., 2022).

Although a growing body of research has examined neighborhood or community environments and depressive symptoms among older adults, much of the existing literature has focused on objective built-environment indicators or selected environmental features, such as greenness, residential density, walkability, neighborhood facilities, or social capital (Lin et al., 2024; Lu et al., 2022; Sarkar et al., 2021; Wang et al., 2024). These studies have provided important evidence, but they may not fully capture how older adults experience their everyday living environments. Perceived community environment may be particularly relevant to depressive symptoms because it reflects older adults’ subjective evaluations of safety, cleanliness, convenience, age-friendliness, service responsiveness, and environmental support in daily life. Compared with objective indicators alone, perceived environmental conditions may be more proximally linked to psychological experience and emotional well-being (Andrews et al., 2021; Mao et al., 2022; Zhang et al., 2019). Moreover, different aspects of perceived community environment may not be equally related to depressive symptoms. For example, perceptions of safety, cleanliness, age-friendly social atmosphere, service responsiveness, and accessibility may reflect different forms of environmental exposure and may operate through different psychosocial pathways (Barnett et al., 2018; Mao et al., 2022). Therefore, examining both the overall perceived community environment and its specific aspects can provide a more nuanced understanding of the community-level correlates of depressive symptoms in later life.

The association between perceived community environment and depressive symptoms may vary across different physical activity (PA). According to the stress-buffering hypothesis, personal or behavioral resources may modify the association between stressful circumstances and mental health outcomes (Cohen and Wills, 1985). Within this framework, an unfavorable perceived community environment may be conceptualized as a chronic contextual stressor, whereas PA may function as a modifiable behavioral resource. PA has been associated with lower depressive symptom scores among older adults and has also been linked to improved physiological functioning, regulated affective states, strengthened self-efficacy and resilience, and facilitated outdoor activity and social contact (Laird et al., 2023; Marques et al., 2020; Nguyen Ho et al., 2023; Pearce et al., 2022; Szuhany et al., 2023; Xue et al., 2025; Zhang et al., 2021). Physically inactive older adults may have more restricted activity spaces and fewer opportunities for social engagement, which may influence how they experience and respond to unfavorable community conditions. However, these potential pathways were not directly examined in the present study. Together, these theoretical and empirical considerations provide a rationale for examining whether the association between perceived community environment and depressive symptoms differs across different PA.

Against this background, several gaps remain in the literature. First, although prior studies have linked community environments and PA separately to depressive symptoms, few have examined whether the association between perceived community environment and depressive symptoms differs across different PA among older adults in China (Barnett et al., 2018; Mao et al., 2022; Zhang et al., 2025). This gap is important because China’s home- and community-based eldercare context makes the community environment especially relevant to older adults’ daily lives and psychological well-being (Krings et al., 2022; Xu et al., 2023; Ying et al., 2024). Second, few studies have examined whether such variation differs across specific aspects of perceived community environment. Because perceived community environment includes multiple dimensions of daily community experience, the associations between specific dimensions and depressive symptoms may vary across different PA. Addressing these gaps may clarify the cross-sectional relationships among perceived community environment, PA, and depressive symptoms and generate hypotheses for future longitudinal and intervention research.

The present study examines the association between perceived community environment and depressive symptoms among older adults in China. Based on the stress-buffering hypothesis, this study further tests whether PA moderates this association. Because perceived community environment includes multiple aspects of community satisfaction, this study also examines whether the moderating effect of PA differs across specific aspects of perceived community environment. Accordingly, the following hypotheses are proposed:

*Hypothesis 1*: Poorer perceived community environment is associated with higher depressive symptoms among older adults.

*Hypothesis 2*: PA moderates the association between perceived community environment and depressive symptoms.

*Hypothesis 3*: The associations between selected dimensions of perceived community environment and depressive symptoms vary across different PA.

**Figure 1:**
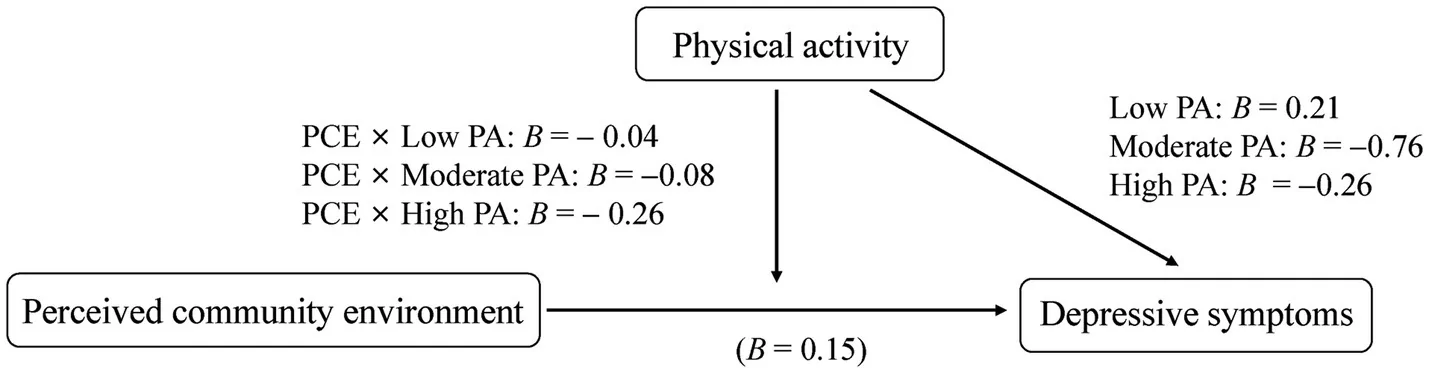
Conceptual framework of the moderating role of physical activity in the association between perceived community environment and depressive symptoms. Values are unstandardized coefficients from the fully adjusted ordinary least-squares linear regression interaction model. Arrows represent statistical associations rather than causal effects.

The conceptual model is presented in Figure 1.

## 2 Materials and methods

### 2.1 Participants

This study used data from the 2023 China Longitudinal Aging Social Survey (CLASS). CLASS is a national, continuous social survey organized by the Institute of Gerontology at Renmin University of China, which targets adults aged 60 years and older in mainland China. The survey adopts a stratified multistage probability sampling design and covers 28 provincial-level administrative units in mainland China, excluding Hong Kong, Macao, Taiwan, Hainan, Xinjiang, and Tibet. CLASS has released individual-level questionnaire data for the 2014, 2016, 2018, 2020, and 2023 waves. The survey collects information on demographic characteristics, health and related services, socioeconomic status, aging plans and social support, psychological well-being, family and children, and daily activities and physical exercise.

The 2023 CLASS included 11,670 respondents. Based on the completeness of key study variables, this study excluded 2,260 respondents with missing data, including those with missing depressive symptoms (*n* = 1,168), self-reported economic status (*n* = 991), marital status (*n* = 77), living alone status (*n* = 4) and survey weights (*n* = 20). The final analytic sample consisted of 9,410 participants. To ensure comparability across the unweighted, community-clustered, and weighted community-clustered analyses, all models were estimated using a common complete-case analytic sample of 9,410 participants.

### 2.2 Independent variables

The independent variable was perceived community environment. In the 2023 CLASS questionnaire, respondents were asked to report their satisfaction with various aspects of their community (village/residential area), including road conditions, exercise facilities, safety, cleanliness, age-friendly social atmosphere, competence of community staff, street lighting, and barrier-free facilities. Each item was rated on a 5-point Likert scale ranging from 1 = very satisfied to 5 = very dissatisfied, with higher scores indicating greater dissatisfaction with the corresponding aspect of the community environment. The eight item scores were summed to generate an overall perceived community environment score ranging from 8 to 40, with higher total scores indicating poorer perceived community environment. In the present analytic sample, the eight-item measure showed good internal consistency, with a Cronbach’s alpha of 0.84.

### 2.3 Dependent variables

The dependent variable was depressive symptoms, measured using the nine-item short form of the Center for Epidemiologic Studies Depression Scale (CES-D-9). This scale assesses respondents’ self-reported depressive symptoms during the past week and has shown acceptable reliability and validity among older adults in China (Silverstein et al., 2006). In the 2023 CLASS questionnaire, respondents were asked to report how often they experienced each symptom during the past week. Each item was rated on a three-point scale: 0 = rarely or none of the time, 1 = some of the time, and 2 = most of the time. Positively worded items, including feeling in good spirits, having a good life, and finding life interesting, were reverse-coded before calculating the total score. The nine-item scores were then summed to generate a total depressive symptom score ranging from 0 to 18, with higher scores indicating more severe depressive symptoms. In the present analytic sample, the scale showed acceptable internal consistency, with a Cronbach’s alpha of 0.70.

### 2.4 Moderating variable

The moderating variable was PA. In the present study, PA was operationalized as weekly PA volume derived from the exercise frequency, duration, and intensity information available in the 2023 CLASS dataset. The 2023 CLASS collected information on exercise frequency, duration, and intensity. Exercise frequency was measured on a nine-category scale, where 1 indicated less than once per month, 2 indicated more than once per month but less than once per week, 3–8 indicated once to six times per week on average respectively, and 9 indicated seven or more times per week. Exercise duration was classified into four categories: 1 = less than 30 min, 2 = 30–59 min, 3 = 60–120 min, and 4 = more than 120 min. Exercise intensity was assessed based on perceived physiological responses during most exercise sessions: 1 = little change in breathing or heart rate, 2 = increased breathing and heart rate with slight sweating, and 3 = shortness of breath, markedly increased heart rate, and heavy sweating.

The nine frequency categories were converted to 0.125, 0.625, 1, 2, 3, 4, 5, 6, and 7 sessions/week, respectively. The four duration categories were assigned 15, 45, 90, and 150 min/session. Metabolic equivalent task values were assigned as 3.3, 4.0, and 8.0 for low-, moderate-, and high-intensity activities, respectively. Following previous research (Li et al., 2024), PA was calculated by multiplying weekly exercise frequency, exercise duration, and metabolic equivalent task value, expressed as MET-min/week. Participants were then categorized into four PA groups: inactive, low PA, moderate PA, and high PA. The classification criteria were as follows: (1) Inactive: exercise frequency <1 session/week or PA < 10 MET-min/week; (2) Low PA: 10 MET-min/week ≤ PA < 600 MET-min/week; (3) Moderate PA: 600 MET-min/week ≤ PA < 3,000 MET-min/week; and (4) High PA: PA ≥ 3,000 MET-min/week (IPAQ Research Committee, 2005). The exact item numbers, original Chinese wording, English translations, and coding procedures are provided in Supplementary Tables S1–S3.

### 2.5 Covariates

Based on prior studies (Agustini et al., 2020; Li et al., 2026; Muhammad and Meher, 2021; Xue et al., 2021), this study included age, gender, residence, marital status, educational level, self-reported economic status, living alone, activities of daily living (ADL) and chronic disease as covariates. Age was entered into the models as a continuous variable. Gender was coded as male = 0 and female = 1; marital status as unmarried = 0 and married = 1; and living alone as no = 0 and yes = 1. Residence was classified as urban = 0 or rural = 1 according to the participant’s reported place of residence. Educational level was categorized as no formal education = 0, junior high school or below = 1, and senior high school or above = 2, with no formal education as the reference group. Self-reported economic status was categorized and coded as low = 0, middle = 1, and high = 2 according to percentile cutoffs, with the low group as the reference category. ADL was coded as a binary variable, with participants reporting limitation in at least one assessed ADL item classified as dependent (coded as 1) and those reporting no limitation classified as independent (coded as 0). Chronic disease was represented by the total number of self-reported chronic conditions.

### 2.6 Statistical analysis

Sample characteristics were summarized using descriptive statistics. Continuous variables were reported as means and standard deviations, and categorical variables were reported as frequencies and percentages. Ordinary least-squares linear regression was used for the primary analysis, with depressive symptoms as a continuous outcome. Perceived community environment was entered into the models as a continuous variable and was mean-centered before analysis.

Three models were estimated: Model 1 included covariates only; Model 2 added perceived community environment and PA; and Model 3 added the interaction term between perceived community environment and PA. PA was entered as a four-category PA variable, with the inactive group as the reference. The same models were fitted for each of the eight community-environment items, with Holm adjustment applied across the eight omnibus interaction tests. Regression assumptions were assessed using residual diagnostic plots, the Ramsey RESET and Breusch–Pagan tests, studentized residuals, Cook’s distance, and VIF/GVIF. Sensitivity analyses used community-clustered HC1 standard errors and weighted models with community-clustered standard errors. Models were also fitted separately for urban and rural participants. All tests were two-sided, with *p* < 0.05. Analyses were conducted in R.

## 3 Results

### 3.1 Sample characteristics

Table 1 compares the participants included and excluded in this analysis. The study included 9,410 older adults, with a mean age of 71.4 years (SD = 6.0). Men accounted for 51.5% of the sample, and most participants were married (83.1%) and did not live alone (91.8%). Regarding educational attainment, 21.5% had no formal education, 66.2% had completed junior high school or below, and 12.4% had completed senior high school or above. Self-reported economic status was distributed across low (30.7%), middle (40.0%), and high (29.3%) groups. More than half of the participants were classified as inactive (53.5%), followed by low PA (30.5%), moderate PA (15.7%), and high PA (0.4%). The mean depressive symptom score was 6.6 (SD = 3.3), and the mean perceived community environment score was 17.0 (SD = 4.3). Participant characteristics stratified by PA are presented in Supplementary Table S4. Several characteristics differed across activity groups, including age, gender, residence, education, marital status, economic status, depressive symptoms, perceived community environment, living alone, and ADL. Because the high PA group included only 37 participants, comparisons involving this group should be interpreted cautiously.

**Table 1:** Sample characteristics.

Variables	Retained participants (*n* = 9,410)	Excluded participants (*n* = 2,260)	*p*-value
Age, years	71.4 (6.0)	70.9 (5.6)	<0.001
Gender			0.085
Male	4,844 (51.5)	1,209 (53.5)	
Female	4,566 (48.5)	1,051 (46.5)	
Residence			<0.001
Urban	5,205 (55.3)	1,410 (62.4)	
Rural	4,205 (44.7)	850 (37.6)	
Education			<0.001
No formal education	2,019 (21.5)	360 (15.9)	
Junior high or below	6,227 (66.2)	1,352 (59.8)	
Senior high or above	1,164 (12.4)	548 (24.2)	
Marital status			<0.001
Married	7,820 (83.1)	1,881 (86.4)	
Unmarried	1,590 (16.9)	295 (13.6)	
Self-reported economic status			<0.001
Low	2,891 (30.7)	270 (24.2)	
Middle	3,761 (40.0)	455 (40.8)	
High	2,758 (29.3)	391 (35.0)	
Physical activity			<0.001
Inactive	5,031 (53.5)	1,416 (62.7)	
Low PA	2,866 (30.5)	574 (25.4)	
Moderate PA	1,476 (15.7)	261 (11.5)	
High PA	37 (0.4)	9 (0.4)	
Depressive symptoms	6.6 (3.3)	5.2 (3.8)	<0.001
PCE	17.0 (4.3)	17.5 (5.0)	<0.001
Living alone			0.313
Yes	774 (8.2)	171 (7.6)	
No	8,636 (91.8)	2,085 (92.4)	
Chronic disease	1.8 (1.4)	1.4 (1.4)	<0.001
ADL			<0.001
Dependent	499 (5.3)	168 (7.4)	
Independent	8,911 (94.7)	2,092 (92.6)	

Continuous variables were compared using Welch’s independent-samples *t*-tests. Categorical variables were compared using Pearson’s Chi-square tests or Fisher’s exact tests, as appropriate. Percentages among excluded participants were calculated using the available non-missing observations for each variable. ADL, activities of daily living; PA, physical activity; PCE, perceived community environment.

### 3.2 Perceived community environment, PA, and depressive symptoms

Table 2 shows the results of the ordinary least-squares multivariable linear regression analyses. In Model 2, poorer perceived community environment was significantly associated with higher depressive symptom scores after adjusting for covariates and PA (*B* = 0.12, 95% CI: 0.11 to 0.14). Compared with inactive participants, those with moderate PA had lower depressive symptom scores (*B* = −0.74, 95% CI: −0.92 to −0.55), whereas those with low PA had slightly higher scores (*B* = 0.21, 95% CI: 0.07 to 0.36). No significant difference was observed for the high PA group. In Model 3, the three interaction terms were jointly statistically significant (*p* < 0.01). The interaction coefficients indicated that, relative to the inactive group, the PCE–depressive symptoms slope was weaker in the low PA group (*B* = −0.04, 95% CI: −0.07 to −0.001), the moderate PA group (*B* = −0.08, 95% CI: −0.12 to −0.03), and the high PA group (B = −0.26, 95% CI: −0.50 to −0.02). Adding the interaction term slightly improved model fit, as indicated by an increase in adjusted *R*^2^ from 0.121 to 0.122 and a decrease in AIC from 48154.12 to 48143.07. Model diagnostics are reported in Supplementary Tables S5, S6. Residual skewness was close to zero across all three ordinary least-squares models, and no observation had a Cook’s distance greater than 1, although a small number of observations had absolute studentized residuals greater than 3. Adjusted GVIF values in the full interaction model ranged from 1.027 to 1.390, indicating no substantial multicollinearity.

**Table 2:** Linear regression of perceived community environment, physical activity, and depressive symptoms among Chinese older adults.

Predictors	Model 1				Model 2				Model 3			
	*B*	SE	95% CI	*p*	*B*	SE	95% CI	*p*	*B*	SE	95% CI	*p*
PCE					0.12	0.01	(0.11, 0.14)	<0.01	0.15	0.01	(0.13, 0.17)	<0.01
PA (ref: inactive)												
Low PA					0.21	0.07	(0.07, 0.36)	<0.01	0.21	0.07	(0.07, 0.36)	<0.01
Moderate PA					−0.74	0.10	(−0.92, −0.55)	<0.01	−0.76	0.10	(−0.95, −0.58)	<0.01
High PA					0.28	0.52	(−0.73, 1.29)	0.59	−0.26	0.59	(−1.41, 0.90)	0.66
Age (years)	0.06	0.01	(0.05, 0.07)	<0.01	0.06	0.01	(0.05, 0.07)	<0.01	0.06	0.01	(0.05, 0.07)	<0.01
Gender (ref: male)												
Female	0.13	0.07	(0.001, 0.27)	<0.05	0.14	0.07	(0.01, 0.27)	0.03	0.14	0.07	(0.01, 0.27)	0.03
Residence (ref: urban)												
Rural	0.63	0.07	(0.49, 0.77)	<0.01	0.42	0.07	(0.28, 0.56)	<0.01	0.41	0.07	(0.27, 0.55)	<0.01
Education (ref: no formal education)												
Junior high or below	−0.51	0.09	(−0.68, −0.34)	<0.01	−0.49	0.09	(−0.65, −0.32)	<0.01	−0.48	0.09	(−0.65, −0.32)	<0.01
Senior high or above	−1.08	0.13	(−1.34, −0.83)	<0.01	−0.92	0.13	(−1.17, −0.67)	<0.01	−0.92	0.13	(−1.17, −0.67)	<0.01
Marital status (ref: unmarried)												
Married	−0.40	0.12	(−0.63, −0.18)	<0.01	−0.39	0.11	(−0.61, −0.17)	<0.01	−0.38	0.11	(−0.61, −0.16)	<0.01
Self-reported economic status (ref: low)												
Middle	−0.61	0.08	(−0.76, −0.45)	<0.01	−0.64	0.08	(−0.80, −0.49)	<0.01	−0.65	0.08	(−0.80, −0.49)	<0.01
High	−0.77	0.09	(−0.95, −0.60)	<0.01	−0.80	0.09	(−0.97, −0.62)	<0.01	−0.79	0.09	(−0.97, −0.62)	<0.01
Living alone (ref: no)												
Yes	0.38	0.15	(0.08, 0.67)	0.01	0.39	0.15	(0.10, 0.68)	0.01	0.39	0.15	(0.10, 0.68)	0.01
ADL (ref: independent)												
Dependent	1.07	0.15	(0.77, 1.37)	<0.01	0.96	0.15	(0.67, 1.25)	<0.01	0.96	0.15	(0.67, 1.25)	<0.01
Chronic disease	0.17	0.02	(0.12, 0.21)	<0.01	0.19	0.02	(0.14, 0.24)	<0.01	0.19	0.02	(0.15, 0.24)	<0.01
Interaction terms												
PCE × low PA									−0.04	0.02	(−0.07, −0.001)	0.04
PCE × moderate PA									−0.08	0.02	(−0.12, −0.03)	<0.01
PCE × high PA									−0.26	0.12	(−0.50, −0.02)	0.04
Adjusted *R*^2^	0.088				0.121				0.122			
Δ Adjusted *R*^2^					0.033				0.001			
AIC	48493.62				48154.12				48143.07			

Values are unstandardized coefficients. In Model 3, the coefficient for the environment score represents its association among inactive participants. Higher PCE scores indicate greater dissatisfaction with the community environment. ADL, activities of daily living; CI, confidence interval; PA, physical activity; PCE, perceived community environment; SE, standard error. Two-sided *p*-values are reported.

Sensitivity analyses are presented in Supplementary Tables S7, S8. After accounting for community clustering, the overall interaction was attenuated and no longer statistically significant (*p* = 0.061), although the interaction for moderate PA remained significant. Similar results were observed in the weighted analysis with community-clustered robust standard errors, in which the overall interaction was also not statistically significant (*p* = 0.096). Residence-stratified analyses in Supplementary Table S9 showed a clearer moderating pattern among urban participants, whereas none of the interaction terms were significant among rural participants. These findings suggest that the primary interaction was sensitive to clustering and weighting and may have been more evident in urban residents.

### 3.3 Item-level moderation and simple-slope analyses

Table 3 presents the item-level moderation analyses for the eight perceived community environment items. Seven omnibus interactions were statistically significant before adjustment for multiple testing: road conditions (*p* = 0.03), safety (*p* < 0.01), cleanliness (*p* < 0.01), age-friendly social atmosphere (*p* = 0.02), street lighting (*p* < 0.01), barrier-free facilities (*p* = 0.02), and competence of community staff (*p* < 0.01). No significant interactions were found for exercise facilities (*p* = 0.20). Supplementary Table S10 reports the corresponding omnibus interaction tests with Holm correction. After correction for multiple testing, significant interactions remained for safety, cleanliness, competence of community staff, and street lighting (Holm-adjusted *p*-values <0.001, 0.006, 0.011, and 0.016, respectively). The interactions for road conditions, age-friendly social atmosphere, barrier-free facilities, and exercise facilities were not statistically significant after correction. These findings suggest that the moderating effect of PA was limited to specific aspects of perceived community environment.

**Table 3:** Item-level interactions between perceived community environment items and physical activity volume group.

Items	Low PA	Moderate PA	High PA
Road conditions	−0.24 (−0.45, −0.03)	−0.27 (−0.53, 0.004)	−1.36 (−3.08, 0.35)
Exercise facilities	0.14 (−0.03, 0.31)	−0.002 (−0.22, 0.21)	−1.04 (−2.64, 0.57)
Safety	−0.13 (−0.32, 0.07)	−0.69 (−0.93, −0.46)	−2.90 (−4.38, −1.41)
Cleanliness	−0.03 (−0.22, 0.15)	−0.42 (−0.65, −0.19)	−1.24 (−2.48, −0.002)
Age-friendly social atmosphere	−0.18 (−0.38, 0.02)	−0.38 (−0.63, −0.13)	−0.28 (−1.60, 1.04)
Competence of community staff	−0.10 (−0.30, 0.09)	−0.48 (−0.72, −0.23)	−0.15 (−1.18, 0.89)
Street lighting	−0.31 (−0.50, −0.13)	−0.16 (−0.39, 0.06)	−1.11 (−2.39, 0.17)
Barrier-free facilities	−0.23 (−0.41, −0.06)	0.02 (−0.20, 0.23)	−0.82 (−1.84, 0.20)

Values are unstandardized interaction coefficients (*B*) with 95% confidence intervals from the fully adjusted ordinary least-squares interaction models. The inactive group was the reference category. Each coefficient represents the difference in the corresponding perceived community environment item slope between that physical activity group and the inactive group. Higher item scores indicate greater dissatisfaction. PA, physical activity.

**Figure 2:**
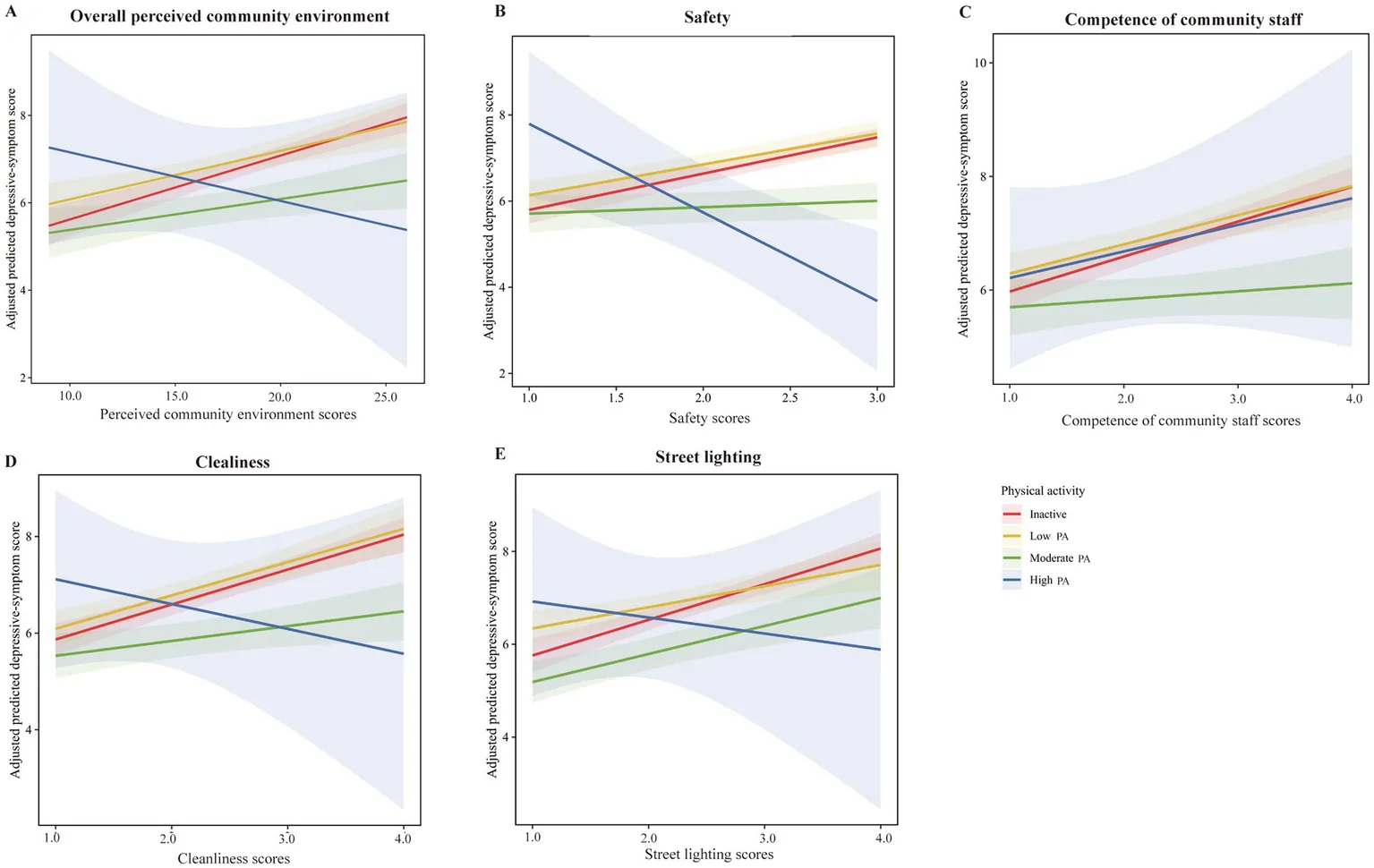
Adjusted associations between perceived community environment and depressive symptoms across physical activity volume groups; Panel **(A)** shows the overall perceived community environment score, and Panels **(B–E)** show the four item-level interactions that remained statistically significant after Holm correction: Safety, competence of community staff, cleanliness, and street lighting. The *x*-axes display the original, uncentered perceived community environment scores. Higher perceived community environment scores indicate greater dissatisfaction. Lines represent adjusted predicted depressive symptom scores, with shaded areas indicating 95% confidence intervals calculated using community-clustered robust standard errors.

As shown in Table 4, greater dissatisfaction with the overall community environment was associated with higher depressive symptom scores in the inactive, low PA, and moderate PA, but not in the high PA. Similar differences in slopes across different PA were observed for safety, cleanliness, competence of community staff, and street lighting. For safety, an inverse association was observed in the high PA. These patterns are also illustrated in Figure 2, although estimates for the high PA should be interpreted cautiously because of the wide confidence intervals.

**Table 4:** Group-specific simple slopes of overall and perceived community environment measures in relation to depressive symptoms.

Items	Inactive	Low PA	Moderate PA	High PA
Overall	0.15 (0.13, 0.17)	0.11 (0.08, 0.14)	0.07 (0.03, 0.11)^a^	−0.11 (−0.35, 0.13)
Safety	0.84 (0.73, 0.96)	0.72 (0.56, 0.87)	0.15 (−0.06, 0.36)^a^^,^^b^	−2.06 (−3.53, −0.58)^a^^,^^b^^,^^c^
Exercise facilities	−0.18 (−0.29, −0.08)	−0.04 (−0.18, 0.09)	−0.18 (−0.37, 0.003)	−1.22 (−2.82, 0.38)
Road conditions	0.72 (0.60, 0.84)	0.48 (0.31, 0.65)	0.45 (0.22, 0.69)	−0.64 (−2.35, 1.07)
Street lighting	0.77 (0.66, 0.88)	0.46 (0.31, 0.60)^a^	0.60 (0.40, 0.80)	−0.35 (−1.62, 0.93)
Barrier-free facilities	0.41 (0.30, 0.52)	0.18 (0.04, 0.32)	0.43 (0.24, 0.62)	−0.41 (−1.42, 0.61)
Cleanliness	0.72 (0.61, 0.84)	0.69 (0.54, 0.84)	0.31 (0.11, 0.51)^a^^,^^b^	−0.51 (−1.75, 0.72)
Age-friendly social atmosphere	0.66 (0.54, 0.78)	0.48 (0.33, 0.64)	0.28 (0.06, 0.50)^a^	0.38 (−0.93, 1.70)
Competence of community staff	0.62 (0.50, 0.73)	0.51 (0.36, 0.67)	0.14 (−0.07, 0.35)^a^^,^^b^	0.47 (−0.56, 1.49)

Values are simple slope estimates (*B*) with 95% confidence intervals within each physical-activity group. Higher PCE scores indicate greater dissatisfaction with the community environment. A 95% confidence interval excluding zero indicates that the within-group slope is statistically significant. Superscripts indicate statistically significant Holm-adjusted pairwise differences between simple slopes. PA, physical activity.

a Significant compared with inactive.

b Significant compared with low PA.

c Significant compared with moderate PA.

## 4 Discussion

This cross-sectional study examined the association between perceived community environment and depressive symptoms among older adults in China and whether this association differed across PA. Three main findings emerged. First, poorer perceived community environment was associated with higher depressive symptom scores. Second, in the primary ordinary least-squares analysis, the association between perceived community environment and depressive symptoms differed across PA. However, the overall interaction was attenuated to non-significance in community-clustered and weighted sensitivity analyses. Third, after Holm correction, significant item-level interactions remained for perceived safety, cleanliness, street lighting, and competence of community staff. Taken together, these findings suggest that the association between perceived community environment and depressive symptoms may vary across PA, although evidence for this variation was not consistent across all model specifications.

First, the finding that poorer perceived community environment was associated with higher depressive symptom scores is consistent with previous research on neighborhood environments and late-life mental health (Barnett et al., 2018; Kou et al., 2024; Lei and Feng, 2021; Lu et al., 2021). For older adults, the community is a central setting of everyday life, providing the immediate context for daily activities, social interaction, access to services, and participation in local life. Subjective perceptions of the community environment may therefore be closely related to feelings of safety, social connectedness, autonomy, and emotional well-being (Robinette et al., 2021; Stephens and Phillips, 2022). Compared with objective built-environment indicators, perceived environment measures may more directly capture how older adults experience their living context and may be more proximally linked to psychological outcomes (Mao et al., 2022; Zhang et al., 2019). When older adults perceive their community as unsafe, unclean, insufficiently age-friendly, or lacking responsive local services, they may experience greater daily stress, weaker social attachment, and a lower sense of environmental support, which may contribute to depressive symptoms (Mao et al., 2022; Zhang et al., 2019). These findings suggest that the community environment is not merely a physical setting for aging but also an important psychosocial context for mental health in later life.

Second, the association between perceived community environment and depressive symptoms differed across PA. This pattern is broadly consistent with the stress-buffering hypothesis, which proposes that personal or behavioral resources may modify the association between stressful circumstances and mental health outcomes (Cohen and Wills, 1985). Simple slope analyses showed that the association between poorer perceived community environment and higher depressive symptoms appeared strongest among inactive participants, and was weaker in the low and moderate PA groups, whereas the estimate for the high PA was not statistically significant. This pattern is also broadly consistent with previous evidence linking PA to a lower risk of depression or fewer depressive symptoms, although the present findings did not indicate a clear dose–response pattern (Pearce et al., 2022; Queiroga et al., 2025). One possible interpretation is that inactive older adults may have more restricted activity spaces and fewer opportunities for outdoor engagement and social contact, which may make unfavorable community conditions more salient in their daily lives. By contrast, PA may be associated with psychosocial resources such as self-efficacy and psychological resilience that are relevant to responses to stressful circumstances (Laird et al., 2023; Szuhany et al., 2023; Zhang et al., 2021). In this sense, PA may operate not only as an individual health behavior but also as a behavioral resource that is relevant to how older adults experience unfavorable community conditions. However, these potential pathways were not directly examined in the present study.

The overall pattern was attenuated in community-clustered and weighted sensitivity analyses, although the interaction for the moderate PA remained statistically significant. Exploratory residence-stratified analyses further showed a clearer pattern among urban participants, whereas none of the interaction terms reached statistical significance among rural participants. These urban–rural patterns may reflect differences in community facilities, activity opportunities, or the role of the local environment in older adults’ daily lives, but these explanations remain speculative because the present study did not directly assess these contextual mechanisms. In addition, the high PA included only 37 participants, accounting for 0.4% of the analytic sample, which limited the precision of the estimate. Overall, these findings suggest that PA may be relevant to variation in the association between perceived community environment and depressive symptoms.

Third, the item-level findings suggest that variation across different PA was concentrated in specific aspects of perceived community environment. After Holm correction, significant interactions remained for perceived safety, cleanliness, street lighting, and competence of community staff. Although these features span both physical and social dimensions of the community environment, they may share a relatively direct relevance to older adults’ everyday community experiences. Perceived safety and street lighting may be relevant to older adults’ confidence in using neighborhood spaces, while cleanliness may contribute to perceptions of order, comfort, and environmental support. Similarly, the competence of community staff may be related to whether local services are perceived as dependable and responsive to residents’ needs. Previous research has linked neighborhood safety and broader physical and social features of age-friendly community environments to depressive symptoms among older adults (Barnett et al., 2018; Lei and Feng, 2021).

One possible explanation is that older adults with different levels of PA may differ in how frequently they encounter and engage with community spaces and services, making these environmental features more or less salient in daily life. PA has been associated with fewer depressive symptoms among older adults (Zhang et al., 2021), while self-efficacy and psychological resilience have been proposed as psychosocial resources that may be relevant to the relationship between PA, stressful circumstances, and mental health (Nguyen Ho et al., 2023; Szuhany et al., 2023). However, these potential pathways were not directly examined in the present study. Overall, the findings suggest that variation across different PA may be more closely related to specific, directly experienced community conditions than to a simple distinction between physical and social environmental domains.

These findings offer preliminary, rather than causal, implications for community-based mental health promotion and healthy aging policy. First, community-based mental health promotion for older adults may consider perceived community environment alongside individual-level screening and treatment. In China’s home- and community-based eldercare context, communities represent important settings for older adults’ daily activities, service use, and social engagement. Routine community assessments may consider incorporating older adults’ perceptions of safety, cleanliness, street lighting, and the competence of community staff to help identify community conditions associated with a greater burden of depressive symptoms.

Second, PA promotion may complement, rather than substitute for, improvements in community conditions, particularly for inactive and low PA older adults. Community-based eldercare and public health services could provide accessible, low-cost, and age-appropriate PA opportunities, such as walking groups, tai chi, square dancing, stretching, and low-intensity group exercise. Such programs may also provide opportunities for regular participation and social interaction. These community conditions may therefore represent potential priorities for community improvement, while appropriate PA programs may provide complementary behavioral and social resources. An integrated approach combining improvements in community conditions, PA promotion, and local eldercare services may offer a useful direction for supporting mental health among older adults in China.

## 5 Limitations

This study has several limitations. First, the cross-sectional design precludes causal inference, and the temporal ordering among perceived community environment, PA, and depressive symptoms cannot be determined. Second, reverse causation is possible, as depressive symptoms may influence both older adults’ evaluations of their community environment and their participation in PA. Third, all three main variables were assessed using self-reported measures and may therefore be affected by recall bias, reporting bias, and common method bias. Future studies should incorporate accelerometers or other wearable devices to obtain more objective estimates of physical activity. In addition, the perceived community environment measure captured only selected aspects of community experience and did not include other potentially relevant dimensions, such as social cohesion, walkability, green space, or service accessibility. Finally, although this study adjusted for several demographic, socioeconomic, and health-related characteristics, residual confounding from unmeasured individual- and community-level factors may remain. Future research should incorporate richer individual- and community-level information and use longitudinal or multilevel designs to strengthen causal inference and generalizability.

## 6 Conclusion

This cross-sectional study found that a poorer perceived community environment was associated with higher depressive symptom scores among older adults in China. In the interaction model, the association appeared strongest among inactive participants and varied across different PA. Item-level analyses further showed that this variation was concentrated in perceived safety, cleanliness, street lighting, and competence of community staff after Holm correction. These findings highlight the relevance of older adults’ subjective evaluations of their communities and suggest that community-based mental health promotion may benefit from integrating improvements in community conditions with accessible and age-appropriate PA opportunities. PA promotion should complement, rather than substitute for, efforts to improve community environments. Given the cross-sectional design and the modest incremental explanatory value of the interaction terms, longitudinal and intervention studies are needed to clarify the temporal relationships among these factors and assess their implications for mental health in later life.

## Data Availability

The datasets presented in this study can be found in online repositories. The names of the repository/repositories and accession number(s) can be found in the article/Supplementary material.
