# Supplementary material for: Perceived community environment and depressive symptoms among older adults in China: a cross-sectional analysis of physical activity as a moderator
Source: Front Psychol. 2026 Aug 24;17:1893833. doi: 10.3389/fpsyg.2026.1893833 (PMC13547465; doi:10.3389/fpsyg.2026.1893833)
Supplement: Supplementary file 1 [file Table_1.docx]

Supplementary Material

[Supplementary Table S1. CLASS questionnaire items assessing perceived community environment	1](#_Toc236337485)

[Supplementary Table S2. CLASS questionnaire items assessing depressive symptoms	2](#_Toc236337486)

[Supplementary Table S3. CLASS questionnaire items assessing physical activity	3](#_Toc236337487)

[Supplementary Table S4. Sample characteristics stratified by physical activity	4](#_Toc236337488)

[Supplementary Table S5. Diagnostic statistics for the ordinary least-squares regression models	5](#_Toc236337489)

[Supplementary Table S6. Multicollinearity assessment for the full interaction model (Model 3)	6](#_Toc236337490)

[Supplementary Table S7. Linear regression with community-clustered robust standard errors	7](#_Toc236337491)

[Supplementary Table S8. Linear regression with weighted community-clustered robust standard errors	8](#_Toc236337492)

[Supplementary Table S9. Residence-stratified linear regression models	9](#_Toc236337493)

[Supplementary Table S10. Holm-adjusted item-level omnibus interaction tests in the primary ordinary least-squares analysis	10](#_Toc236337494)

# Supplementary Table S1. CLASS questionnaire items assessing perceived community environment

| **Construct** | **CLASS item number** | **Original Chinese wording** |
| --- | --- | --- |
| Road conditions | B24_1 | 您对本社区（村居）内的道路情况的满意程度是？  How satisfied are you with the road conditions in your community? |
| Exercise facilities | B24_2 | 您对本社区（村居）内的健身/活动场所的满意程度是？  How satisfied are you with the exercise/activity facilities in your community? |
| Safety | B24_3 | 您对本社区（村居）内的治安环境的满意程度是？  How satisfied are you with the safety conditions in your community? |
| Cleanliness | B24_4 | 您对本社区（村居）内的环境卫生的满意程度是？  How satisfied are you with the environmental cleanliness in your community? |
| Age-friendly social atmosphere | B24_5 | 您对本社区（村居）内的尊老敬老氛围的满意程度是？  How satisfied are you with the age-friendly social atmosphere in your community? |
| Competence of community staff | B24_6 | 您对本社区（村居）内居委会（村委会）工作人员能力的满意程度是？  How satisfied are you with the competence of community staff? |
| Street lighting | B24_7 | 您对本社区（村居）内的道路/街道照明的满意程度是？  How satisfied are you with the street lighting conditions in your community? |
| Barrier-free facilities | B24_8 | 您对本社区（村居）内的无障碍设施的满意程度是？  How satisfied are you with the accessibility of barrier-free facilities in your community? |

Notes: All items were rated on a 5-point scale: 1 = 非常满意 (very satisfied), 2 = 比较满意 (satisfied), 3 = 一般 (neutral), 4 = 比较不满意 (dissatisfied), and 5 = 非常不满意 (very dissatisfied). The eight items were summed to generate the perceived community environment score (possible range, 8−40), with higher scores indicating greater dissatisfaction with the community environment. CLASS, China Longitudinal Aging Social Survey.

# Supplementary Table S2. CLASS questionnaire items assessing depressive symptoms

| **CLASS item number** | **Original Chinese wording** |
| --- | --- |
| E2_1 | 过去一周您觉得自己心情很好吗？  During the past week, did you feel that you were in a good mood? |
| E2_2 | 过去一周您觉得孤单吗？  During the past week, did you feel lonely? |
| E2_3 | 过去一周您觉得心里很难过吗?  During the past week, did you feel sad? |
| E2_4 | 过去一周您觉得自己的日子过得很不错吗？  During the past week, did you feel that you were enjoying life? |
| E2_5 | 过去一周您觉得不想吃东西吗？  During the past week, did you feel that you did not want to eat? |
| E2_6 | 过去一周您睡眠不好吗？  During the past week, did you sleep poorly? |
| E2_7 | 过去一周您觉得自己不中用了吗？  During the past week, did you feel that you were useless? |
| E2_8 | 过去一周您觉得自己没事可做吗？  During the past week, did you feel that you had nothing to do? |
| E2_9 | 过去一周您觉得生活中有很多乐趣（有意思的事情）吗？  During the past week, did you feel that there were many enjoyable or interesting things in your life? |

Notes: All items were originally coded as 1 = rarely or none of the time, 2 = some of the time, and 3 = most of the time. Responses were recoded from 1–3 to 0–2 before summation. The positively worded items E2_1, E2_4, and E2_9 were reverse-scored (0 = 2, 1 = 1, and 2 = 0). Responses coded as 9 indicated no response and were treated as missing. Item scores were summed, with higher total scores indicating more depressive symptoms (possible range, 0–18).

# Supplementary Table S3. CLASS questionnaire items assessing physical activity

| **Construct** | **CLASS**  **item number** | **Item wording** | **Response options coding** |
| --- | --- | --- | --- |
| Physical activity frequency | G2 | 您参加体育锻炼的频率是：  How often do you participate in physical exercise? | 1 = 平均每月不足1次 (less than once per month) 2 = 平均每月1次以上，但每周不足1次 (at least once per month but less than once per week) 3 = 平均每周1次 (once per week) 4 = 平均每周2次 (twice per week) 5 = 平均每周3次 (three times per week) 6 = 平均每周4次 (four times per week) 7 = 平均每周5次 (five times per week) 8 = 平均每周6次 (six times per week) 9 = 平均每周7次及以上 (seven or more times per week) |
| Physical activity duration | G3 | 您平均每次体育锻炼的时间是：  What is the average duration of each physical exercise session? | 1 = 不足30分钟 (less than 30 minutes) 2 = 30–59分钟 (30–59 minutes) 3 = 60–120分钟 (60–120 minutes) 4 = 120分钟以上 (more than 120 minutes) |
| Physical activity intensity | G4 | 您在参加体育锻炼时，多数时间身体感觉比平时：  During physical exercise, which of the following best describes your usual physical response? | 1 = 呼吸、心跳变化不大 (little or no change in breathing or heart rate) 2 = 呼吸、心跳加快，微微出汗 (faster breathing and heart rate, with slight sweating) 3 = 呼吸急促，心跳明显加快，出汗较多 (shortness of breath, markedly increased heart rate, and heavy sweating) |

Notes: Physical activity was derived from frequency, duration, and intensity responses. Frequency and duration categories were converted to midpoint values, and intensity categories were assigned MET values. Participants were then classified into the inactive, low, moderate, and high activity-volume groups according to the study coding procedure.

# Supplementary Table S4. Sample characteristics stratified by physical activity

| **Variables** | **Inactive** | **Low PA** | **Moderate PA** | **High PA** |
| --- | --- | --- | --- | --- |
| **Age** | 72.1 (6.2) | 71.0 (5.9)^a^ | 70.1 (5.1)^ab^ | 68.5 (3.9)^abc^ |
| **Gender** |  |  |  |  |
| Male | 2,584 (51.4) | 1,525 (53.2) | 718 (48.6)^b^ | 17 (45.9) |
| Female | 2,447 (48.6) | 1,341 (46.8) | 758 (51.4)^b^ | 20 (54.1) |
| **Residence** |  |  |  |  |
| Urban | 2,510 (49.9) | 1,605 (56.0)^a^ | 1,061 (71.9)^ab^ | 29 (78.4)^ab^ |
| Rural | 2,521 (50.1) | 1,261 (44.0)^a^ | 415 (28.1)^ab^ | 8 (21.6)^ab^ |
| **Education level** |  |  |  |  |
| No formal education | 1,291 (25.7) | 569 (19.9)^a^ | 156 (10.6)^ab^ | 3 (8.1)^a^ |
| Junior high or below | 3,233 (64.3) | 1,953 (68.1)^a^ | 1,014 (68.7)^a^ | 27 (73.0) |
| Senior high or above | 507 (10.1) | 344 (12.0)^a^ | 306 (20.7)^ab^ | 7 (18.9) |
| **Marital status** |  |  |  |  |
| Married | 4,049 (80.5) | 2,442 (85.2)^a^ | 1,294 (87.7)^a^ | 35 (94.6) |
| Unmarried | 982 (19.5) | 424 (14.8)^a^ | 182 (12.3)^a^ | 2 (5.4) |
| **Self-reported economic status** | |  |  |  |
| Low | 1,653 (32.9) | 878 (30.6)^a^ | 350 (23.7)^ab^ | 10 (27.0) |
| Middle | 2,049 (40.7) | 1,011 (35.3)^a^ | 679 (46.0)^ab^ | 22 (59.5) ^b^ |
| High | 1,329 (26.4) | 977 (34.1)^a^ | 447 (30.3)^ab^ | 5 (13.5) ^b^ |
| **Depressive symptoms** | 6.8 (3.3) | 6.8 (3.2) | 5.5 (3.3) ^ab^ | 6.2 (3.7) |
| **Perceived community environment** | 17.1 (4.3) | 17.1 (4.2) | 16.4 (4.3) ^ab^ | 14.7 (4.2) ^abc^ |
| **Living alone** |  |  |  |  |
| Yes | 461 (9.2) | 220 (7.7) | 92 (6.2)^a^ | 1 (2.7) |
| No | 4,570 (90.8) | 2,646 (92.3) | 1,384 (93.8)^a^ | 36 (97.3) |
| **Chronic disease** | 1.8 (1.5) | 1.9 (1.4) | 1.8 (1.4) | 2.3 (1.7) |
| **ADL** |  |  |  |  |
| Dependent | 338 (6.7) | 134 (4.7)^a^ | 27 (1.8)^ab^ | 0 (0.0) |
| Independent | 4,693 (93.3) | 2,732 (95.3)^a^ | 1449 (98.2)^ab^ | 37 (100.0) |

Notes: Continuous variables were compared using Welch’s one-way ANOVA, followed by Holm-adjusted pairwise Welch t tests. Categorical variables were compared using Pearson’s chi-square test or Fisher’s exact test, as appropriate, followed by Holm-adjusted pairwise comparisons. ^a^ Significantly different from the inactive group; ^b^ significantly different from the low activity-volume group; ^c^ significantly different from the moderate activity-volume group. ADL, activities of daily living.

# Supplementary Table S5. Diagnostic statistics for the ordinary least-squares regression models

| **Model** | **Intercept** | **Residual SD** | **N** | **Residual df** | **Residual skewness** | **Maximum \|studentized residual\|** | **No. with \|studentized residual\| > 3** | **Maximum Cook's distance** | **No. with Cook's distance > 1** | **Residual excess kurtosis** | **Breusch–Pagan P value** | **Ramsey RESET P value** |
| --- | --- | --- | --- | --- | --- | --- | --- | --- | --- | --- | --- | --- |
| Model 1 | 2.692 | 3.180 | 9,410 | 9398 | −0.028 | 3.473 | 6 | 0.002 | 0 | −0.527 | <0.001 | <0.001 |
| Model 2 | 2.773 | 3.123 | 9,410 | 9394 | 0.020 | 3.672 | 9 | 0.010 | 0 | −0.469 | <0.001 | <0.001 |
| Model 3 | 2.762 | 3.121 | 9,410 | 9391 | 0.024 | 3.707 | 8 | 0.048 | 0 | −0.471 | <0.001 | <0.001 |

Notes: Model 1 included covariates only; Model 2 additionally included perceived community environment and physical activity; Model 3 additionally included the perceived community environment × physical activity interaction terms. Residual skewness is the adjusted Fisher-Pearson coefficient. Studentized residuals are externally studentized. Thresholds were used for screening only; no observation had Cook's distance > 1.

# Supplementary Table S6. Multicollinearity assessment for the full interaction model (Model 3)

| **Predictor** | **GVIF** | **df** | **GVIF^(1/(2×df))** |
| --- | --- | --- | --- |
| Perceived community environment | 1.933 | 1 | 1.390 |
| Physical activity | 1.406 | 3 | 1.058 |
| Age | 1.266 | 1 | 1.125 |
| Gender | 1.055 | 1 | 1.027 |
| Marital status | 1.755 | 1 | 1.325 |
| Education | 1.264 | 2 | 1.060 |
| Self-reported economic status | 1.215 | 2 | 1.050 |
| Living alone | 1.594 | 1 | 1.263 |
| ADL | 1.078 | 1 | 1.038 |
| Chronic disease | 1.085 | 1 | 1.042 |
| Residence | 1.191 | 1 | 1.091 |
| PCE × physical activity | 2.497 | 3 | 1.165 |

Notes: GVIF, generalized variance inflation factor; PCE, perceived community environment; ADL, activities of daily living. For multi-degree-of-freedom terms, GVIF^(1/(2×df)) was used to facilitate comparison across predictors. Adjusted GVIF values ranged from 1.027 to 1.390.

# Supplementary Table S7. Linear regression with community-clustered robust standard errors

|  | **Model 1** | **Model 2** | **Model 3** |
| --- | --- | --- | --- |
| **Age** | 0.06 (0.04, 0.08) | 0.06 (0.04, 0.08) | 0.06 (0.05, 0.08) |
| **Gender (ref. male)** |  |  |  |
| Female | 0.13 (−0.001, 0.27) | 0.14 (0.01, 0.27) | 0.14 (0.01, 0.28) |
| **Residence (ref. urban)** |  |  |  |
| Rural | 0.63 (0.28, 0.98) | 0.42 (0.08, 0.75) | 0.41 (0.07, 0.75) |
| **Education (ref. no formal education)** |  |  |  |
| Junior high or below | −0.51 (−0.75, −0.27) | −0.49 (−0.72, −0.26) | −0.48 (−0.71, −0.25) |
| Senior high or above | −1.08 (−1.49, −0.68) | −0.92 (−1.31, −0.52) | −0.92 (−1.31, −0.53) |
| **Marital status (ref. unmarried)** |  |  |  |
| Married | −0.40 (−0.63, −0.17) | −0.39 (−0.61, −0.16) | −0.38 (−0.61, −0.16) |
| **Self-reported economic status (ref. low)** |  |  |  |
| Middle | −0.60 (−0.85, −0.35) | −0.64 (−0.88, −0.40) | −0.65 (−0.89, −0.40) |
| High | −0.77 (−1.09, −0.46) | −0.80 (−1.10, −0.50) | −0.79 (−1.09, −0.50) |
| **Living alone (ref. no)** |  |  |  |
| Yes | 0.38 (0.09, 0.67) | 0.39 (0.11, 0.67) | 0.39 (0.11, 0.66) |
| **ADL (ref. independent)** |  |  |  |
| Dependent | 1.07 (0.75, 1.39) | 0.96 (0.64, 1.28) | 0.96 (0.64, 1.28) |
| **Chronic disease** | 0.17 (0.05, 0.28) | 0.19 (0.08, 0.30) | 0.19 (0.09, 0.30) |
| **PCE** |  | 0.12 (0.09, 0.15) | 0.15 (0.11, 0.18) |
| **Physical activity (ref. inactive)** |  |  |  |
| Low |  | 0.21 (−0.09, 0.51) | 0.21 (−0.09, 0.52) |
| Moderate |  | −0.74 (−1.13, −0.34) | −0.76 (−1.16, −0.37) |
| High |  | 0.28 (−1.12, 1.68) | −0.26 (−1.62, 1.11) |
| **PCE × physical activity** |  |  |  |
| PCE × low PA |  |  | −0.04 (−0.10, 0.03) |
| PCE × moderate PA |  |  | −0.08 (−0.14, −0.01) |
| PCE × high PA |  |  | −0.26 (−0.53, 0.02) |
| Adjusted R² | 0.088 | 0.121 | 0.122 |
| Δ Adjusted R² |  | 0.033 | 0.001 |
| AIC | 48493.62 | 48154.12 | 48143.07 |

Notes: Values are unstandardized regression coefficients with 95% confidence intervals. Higher PCE scores indicate greater dissatisfaction with the perceived community environment. ADL: activity of daily living; PCE: perceived community environment.

# Supplementary Table S8. Linear regression with weighted community-clustered robust standard errors

|  | **Model 1** | **Model 2** | **Model 3** |
| --- | --- | --- | --- |
| **Age** | 0.07 (0.05, 0.09) | 0.07 (0.05, 0.09) | 0.07 (0.05, 0.09) |
| **Gender (ref. male)** |  |  |  |
| Female | 0.11 (−0.03, 0.26) | 0.13 (−0.02, 0.27) | 0.13 (−0.02, 0.27) |
| **Residence (ref. urban)** |  |  |  |
| Rural | 0.63 (0.26, 1.00) | 0.43 (0.07, 0.79) | 0.43 (0.07, 0.79) |
| **Education (ref. no formal education)** |  |  |  |
| Junior high or below | −0.49 (−0.75, −0.23) | −0.47 (−0.72, −0.22) | −0.46 (−0.71, −0.21) |
| Senior high or above | −1.03 (−1.46, −0.59) | −0.87 (−1.29, −0.44) | −0.86 (−1.29, −0.44) |
| **Marital status (ref. unmarried)** |  |  |  |
| Married | −0.50 (−0.75, −0.25) | −0.48 (−0.72, −0.23) | −0.48 (−0.72, −0.23) |
| **Self-reported economic status (ref. low)** |  |  |  |
| Middle | −0.69 (−0.95, −0.43) | −0.71 (−0.96, −0.45) | −0.71 (−0.96, −0.45) |
| High | −0.84 (−1.17, −0.51) | −0.86 (−1.18, −0.55) | −0.85 (−1.17, −0.54) |
| **Living alone (ref. no)** |  |  |  |
| Yes | 0.28 (−0.05, 0.62) | 0.33 (0.007, 0.65) | 0.31 (−0.01, 0.64) |
| **ADL (ref. independent)** |  |  |  |
| Dependent | 1.37 (0.97, 1.76) | 1.25 (0.86, 1.65) | 1.25 (0.85, 1.65) |
| **Chronic disease** | 0.16 (0.03, 0.29) | 0.18 (0.06, 0.30) | 0.19 (0.07, 0.30) |
| **PCE** |  | 0.11 (0.08, 0.14) | 0.14 (0.10, 0.18) |
| **Physical activity (ref. inactive)** |  |  |  |
| Low PA |  | 0.23 (−0.10, 0.56) | 0.23 (−0.09, 0.56) |
| Moderate PA |  | −0.74 (−1.15, −0.33) | −0.76 (−1.17, −0.35) |
| High PA |  | 0.56 (−0.78, 1.90) | 0.03 (−1.36, 1.42) |
| **PCE × physical activity** |  |  |  |
| PCE × low PA |  |  | −0.04 (−0.11, 0.03) |
| PCE × moderate PA |  |  | −0.07 (−0.14, −0.003) |
| PCE × high PA |  |  | −0.25 (−0.53, 0.04) |
| Adjusted R² | 0.081 | 0.111 | 0.112 |
| Δ Adjusted R² |  | 0.030 | 0.001 |
| AIC | 49804.82 | 49501.00 | 49490.16 |

Notes: Values are unstandardized regression coefficients with 95% confidence intervals. Higher PCE scores indicate greater dissatisfaction with the perceived community environment. ADL: activity of daily living; PCE: perceived community environment.

# Supplementary Table S9. Residence-stratified linear regression models

| **Variable** | **Urban (n = 5,205)** | | | **Rural (n = 4,205)** | | |
| --- | --- | --- | --- | --- | --- | --- |
|  | **Model 1** | **Model 2** | **Model 3** | **Model 1** | **Model 2** | **Model 3** |
| **Age** | 0.07 (0.05, 0.08) | 0.06 (0.05, 0.08) | 0.07 (0.05, 0.08) | 0.05 (0.03, 0.07) | 0.05 (0.04, 0.07) | 0.05 (0.03, 0.07) |
| **Gender (ref. male)** |  |  |  |  |  |  |
| Female | 0.13 (−0.05, 0.31) | 0.16 (−0.02, 0.34) | 0.17 (−0.01, 0.34) | 0.12 (−0.07, 0.32) | 0.11 (−0.08, 0.30) | 0.11 (−0.08, 0.30) |
| **Education (ref. no formal education)** |  |  |  |  |  |  |
| Junior high or below | −0.44 (−0.70, −0.17) | −0.35 (−0.61, −0.08) | −0.33 (−0.59, −0.07) | −0.58 (−0.80, −0.37) | −0.60 (−0.81, −0.39) | −0.60 (−0.81, −0.38) |
| Senior high or above | −1.06 (−1.39, −0.73) | −0.85 (−1.17, −0.52) | −0.84 (−1.16, −0.51) | −0.60 (−1.12, −0.09) | −0.47 (−0.98, 0.04) | −0.48 (−0.99, 0.03) |
| **Marital status (ref. unmarried)** |  |  |  |  |  |  |
| Married | −0.65 (−0.97, −0.32) | −0.60 (−0.92, −0.29) | −0.58 (−0.90, −0.26) | −0.10 (−0.42, 0.22) | −0.11 (−0.42, 0.20) | −0.12 (−0.43, 0.20) |
| **Self-reported economic status (ref. low)** |  |  |  |  |  |  |
| Middle | −0.45 (−0.68, −0.22) | −0.37 (−0.60, −0.14) | −0.38 (−0.61, −0.15) | −0.78 (−0.99, −0.57) | −0.90 (−1.11, −0.69) | −0.89 (−1.09, −0.68) |
| High | −0.82 (−1.06, −0.58) | −0.75 (−0.99, −0.51) | −0.73 (−0.97, −0.50) | −0.45 (−0.73, −0.17) | −0.58 (−0.86, −0.30) | −0.56 (−0.84, −0.28) |
| **Living alone (ref. no)** |  |  |  |  |  |  |
| Yes | 0.11 (−0.32, 0.53) | 0.14 (−0.27, 0.56) | 0.14 (−0.28, 0.56) | 0.70 (0.29, 1.10) | 0.70 (0.30, 1.10) | 0.69 (0.29, 1.09) |
| **ADL (ref. independent)** |  |  |  |  |  |  |
| Dependent | 1.15 (0.76, 1.54) | 1.03 (0.65, 1.41) | 1.02 (0.64, 1.41) | 1.01 (0.55, 1.47) | 0.91 (0.46, 1.37) | 0.91 (0.46, 1.37) |
| **Chronic disease** | 0.08 (0.02, 0.14) | 0.11 (0.05, 0.17) | 0.13 (0.06, 0.19) | 0.32 (0.25, 0.39) | 0.33 (0.26, 0.40) | 0.33 (0.26, 0.40) |
| **PCE** | — | 0.13 (0.10, 0.15) | 0.18 (0.14, 0.21) | — | 0.11 (0.09, 0.13) | 0.12 (0.09, 0.14) |
| **Physical activity (ref. inactive)** |  |  |  |  |  |  |
| Low PA | — | 0.18 (−0.02, 0.38) | 0.17 (−0.03, 0.37) | — | 0.27 (0.07, 0.48) | 0.27 (0.07, 0.48) |
| Moderate PA | — | −0.85 (−1.08, −0.61) | −0.87 (−1.10, −0.64) | — | −0.66 (−0.98, −0.34) | −0.65 (−0.97, −0.33) |
| High PA | — | 0.73 (−0.45, 1.90) | 0.05 (−1.23, 1.33) | — | −1.60 (−3.70, 0.50) | −0.55 (−2.99, 1.90) |
| **Interaction terms** |  |  |  |  |  |  |
| PCE × low PA | — | — | −0.05 (−0.10, −0.002) | — | — | −0.02 (−0.07, 0.02) |
| PCE × moderate PA | — | — | −0.14 (−0.19, −0.08) | — | — | 0.02 (−0.06, 0.09) |
| PCE × high PA | — | — | −0.40 (−0.67, −0.13) | — | — | 0.47 (−0.10, 1.03) |
| R² | 0.073 | 0.109 | 0.114 | 0.084 | 0.116 | 0.116 |
| Adjusted R² | 0.071 | 0.106 | 0.111 | 0.082 | 0.113 | 0.113 |
| Δ adjusted R² | — | 0.035 | 0.005 | — | 0.030 | <0.001 |

Notes: Values are unstandardized regression coefficients (B) with 95% confidence intervals. PCE, perceived community environment; PA, physical activity; ADL, activities of daily living. Residence was not included as a covariate because analyses were stratified by residence.

# Supplementary Table S10. Holm-adjusted item-level omnibus interaction tests in the primary ordinary least-squares analysis

| **Community-environment item** | **F** | **df1** | **df2** | **Raw p** | **Holm-adjusted p** |
| --- | --- | --- | --- | --- | --- |
| Road conditions | 3.075 | 3 | 9391 | 0.026 | 0.071 |
| Exercise facilities | 1.568 | 3 | 9391 | 0.195 | 0.195 |
| Safety | 15.342 | 3 | 9391 | <0.001 | **<0.001** |
| Cleanliness | 5.584 | 3 | 9391 | <0.001 | **0.006** |
| Age-friendly social atmosphere | 3.372 | 3 | 9391 | 0.018 | 0.071 |
| Competence of community staff | 4.971 | 3 | 9391 | 0.002 | **0.011** |
| Street lighting | 4.613 | 3 | 9391 | 0.003 | **0.016** |
| Barrier-free facilities | 3.246 | 3 | 9391 | 0.021 | 0.071 |

Notes**:** Each item-level omnibus test jointly evaluated the three perceived community environment × physical activity interaction coefficients. Holm adjustment was applied across the eight item-level tests. Bold values indicate Holm-adjusted P < 0.05
